# Immune Checkpoint Inhibition in Oesophago-Gastric Carcinoma

**DOI:** 10.3390/ph14020151

**Published:** 2021-02-12

**Authors:** Anica Högner, Peter Thuss-Patience

**Affiliations:** Campus Virchow-Klinikum, Medizinische Klinik m.S. Hämatologie, Onkologie und Tumorimmunologie, Charité—Universitätsmedizin Berlin, Corporate Member of Freie Universität Berlin, Humboldt-Universität zu Berlin, and Berlin Institute of Health, Augustenburger Platz 1, 13353 Berlin, Germany; anica.hoegner@charite.de

**Keywords:** gastrointestinal cancer, oesophageal cancer, stomach cancer, immune checkpoint inhibitors, programmed death receptor, nivolumab, pembrolizumab

## Abstract

Immune checkpoint inhibitors enrich the therapeutic landscape in oesophago-gastric carcinoma. With regard to oesophageal squamous cell carcinoma (ESCC), the selective PD-1 (programmed cell death receptor 1)-inhibitor nivolumab improves disease-free survival in the adjuvant therapy setting (CHECKMATE-577). In first-line treatment, ESCC patients (pts) benefit in overall survival (OS) from the PD-1-inhibitor pembrolizumab in combination with chemotherapy (KEYNOTE-590). In the second-line setting, nivolumab (ATTRACTION-03) and pembrolizumab (KEYNOTE-181) demonstrate a benefit in OS compared with chemotherapy. These data resulted in the approval of nivolumab for the second-line treatment of advanced ESCC pts regardless of PD-L1 (programmed cell death ligand 1) status in Europe, Asia, and the USA, and pembrolizumab for pts with PD-L1 CPS (combined positivity score) ≥ 10 in Asia and the USA. Further approvals can be expected. In gastro-oesophageal junction and gastric cancer, the addition of nivolumab to chemotherapy in first-line treatment improves OS in pts with advanced disease with PD-L1 CPS ≥ 5 (CHECKMATE-649). Additionally, pembrolizumab was non-inferior to chemotherapy for OS in PD-L1 CPS ≥ 1 pts (KEYNOTE-062). In third-line treatment, nivolumab shows benefits in OS regardless of PD-L1 expression (ATTRACTION-02) with approval in Asia, and pembrolizumab prolonged the duration of response in PD-L1 positive pts (KEYNOTE-059) with approval in the USA. We discuss the recent results of the completed phase II and III clinical trials.

## 1. Introduction

Oesophageal cancer causes around 572,000 new cases globally and ranks 9th out of the 10 most common new cancer cases worldwide. About 509,000 deaths were estimated globally in 2018 [1]. In Europe, the incidence accounts for 53,000 new cases (40,700 men vs. 12,300 women) and 45,100 deaths (34,900 men vs. 10,100 women). There is a consistent decline in the mortality rates of oesophageal cancer mortality in men (from 4.5 in 2015 to 3.2/100,000), whereas the mortality rates of women stay stable (from 1.1 in 2015 to 1.2/100,000) [2]. Worldwide, approximately 90% of all oesophageal cancer cases account for oesophageal squamous cell carcinoma (ESCC) and 10% for oesophageal adenocarcinoma (EAC), respectively [3]. In Europe, broad regional differences exist in incidental rates dependent on the histological subtype with increasing EAC cases in most countries, while ESCC trends decrease or stabilize [4]. Histological differentiation is relevant for individual therapy as both subgroups represent a separate disease with separate molecular patterns and risk factors [3].

In contrast to the low incidence of oesophageal cancer, stomach cancer ranks sixth place among common cancer cases worldwide (incidence ~1,033,000 cases), resulting in approximately 780,000 deaths. Globally, stomach cancer is the third most common cause of death from cancer [1]. Within Europe, the incidence estimate is 81,600 cases in men and 51,500 in women. Gastric cancer causes 62,000 deaths in men and 40,300 deaths in women, which places gastric cancer as the fifth most common cancer in European men [2]. Histological classification comprises three main subtypes as defined by Lauren’s classification: intestinal type (~54%), diffuse type (~32%), and mixed type (15%) [4].

In many European countries, medical treatment does not depend so much on the localisation of the tumour (oesophageal versus gastric) but more on the histology (adenocarcinoma versus squamous cell carcinoma). This approach is supported by molecular analyses, which show that EAC is different from ESCC, but similar to gastric cancer [4]. In contrast to this, some global clinical trials included both histological subtypes within one trial, so that we follow the discussion of the results according to their division in this review.

At the time of diagnosis, frequently gastrointestinal cancers have already metastasised. Innovative multimodal therapy regimes have successfully improved the prognosis of oesophageal and stomach cancer within the last years. In gastro-oesophageal cancer, the latest improvements in systemic treatment were the introduction of ramucirumab and TAS 102 in the palliative setting [5,6]. There is still a high medical need for further improvements. Inhibition of immune checkpoints is changing the treatment paradigms of many solid tumors and has also been investigated in gastro-oesophageal cancer. Cancer cells have the ability to evade the anti-tumor immune response by expressing PD-L1 (programmed cell death ligand 1) on the cell surface which inhibits the cytotoxic T-cells through binding and blockade of the T-cell receptor PD-1 (programmed cell death receptor 1). By overexpression of PD-L1 on their surface or inducing PD-L1 expression on immune cells, cancer cells exploit the PD-1/PD-L1 pathway to further promote immunoevasion. Furthermore, cancer cell-mediated upregulation of CTLA-4 (anti-cytotoxic T-lymphocyte-associated antigen 4) on T-cells enhances the recruitment of immunosuppressive T-cells and constitutes a co-inhibitory pathway to elude host immune responses.

Antibodies against the checkpoint proteins PD-1/PD-L1/2/CTLA-4 release this anti-tumor brake and reactivate protective T-cell activity [7]. Currently, phase II and phase III trials demonstrate a benefit in the overall survival of patients suffering from oesophago-gastric cancers by immune checkpoint inhibition in different therapy lines (Table 1).

## 2. Molecular Interaction of Checkpoint Inhibitors in Oesophago-Gastric Cancer

Immune checkpoint inhibition is in advance not only in melanoma, renal cell cancer, bladder cancer, and non small cell lung cancer (NSCLC), but also in gastrointestinal cancers. Regulatory T-cells depend on the activity of CTLA-4, PD-1, and PD-L1 to induce immunosuppression [8]. Cancer cells create an immunosuppressive environment. Through overexpression of PD-L1 on the cancer cell surface or inducing PD-L1/CTLA-4 expression on immune cells, cancer cells use the PD-1/PD-L1 and CTLA-4 pathway resulting in immune escape and tumor growth. Drugs that block the checkpoint proteins PD-1, PD-L1, CTLA-4 reactivate and enhance antitumoral T-cell-mediated immune response by blocking these inhibitory signals [9,10,11,12,13].

**Figure 1:**
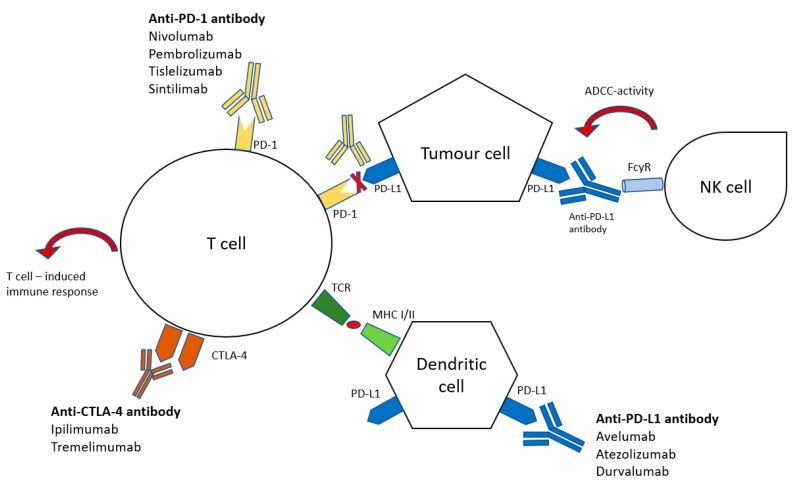
Functional interaction of immune system and tumour cells in checkpoint inhibition (adapted from Taieb et al., 2018 [13].) Blocking checkpoint proteins, PD-1 (programmed death receptor-1), PD-L1 (programmed death receptor ligand-1), CTLA-4 (anti-cytotoxic T-lymphocyte-associated antigen 4), amplify T-cell immune response against tumour cells by blocking inhibitory signals of tumour cells. NK—natural killer, FcyR—Fc receptors for IgG, ADCC—antibody-dependent cellular cytotoxicity, MHC I/II—major histocompatibility complex, TCR—T-cell receptor.

A distinction is made between three groups of selective monoclonal antibodies acting as immune checkpoint inhibitors in gastrointestinal cancers, dependent on the target of action: nivolumab/pembrolizumab/tislelizumab/sintilimab targetting the programmed death receptor-1, (“PD-1”-blockade), atezolizumab/avelumab/durvalumab targetting the programmed death receptor ligand 1 (“PD-L1”-blockade), ipilimumab/tremelimumab targeting CTLA-4 (cytotoxic T-cell associated antigen-4, “CTLA-4”-blockade). Anti-PD-1-antibodies specifically bind the PD-1 protein on the surface of T-cells and therefore prevent the link of PD-1 and PD-L1 on the tumour cell. Anti-PD-L1 antibodies bind to PD-L1 proteins, inhibit the link between PD-1 and PD-L1, and may mediate antibody-dependent cellular cytotoxicity of natural killer cells (via Fc receptors for IgG) as well as activation of dendritic cells which in turn activate anti-tumour T-cell induced immune response. The CTLA-4 protein, physiologically expressed on the surface of immune cells, is involved in peripheral tolerance and prevention of autoimmunity. By upregulation of the CTLA-4 proteins, cancer cells exploit this co-inhibitory pathway of the immune system to further suppress immune response. Specific antibodies against this CTLA-4 inhibit this stimulatory interaction and therefore reactivate T-cell induced immune response [7] (Figure 1).

## 3. Immune Checkpoint Inhibition in the Treatment of Oesophageal Cancer

### 3.1. Curative Setting

**Figure 2:**
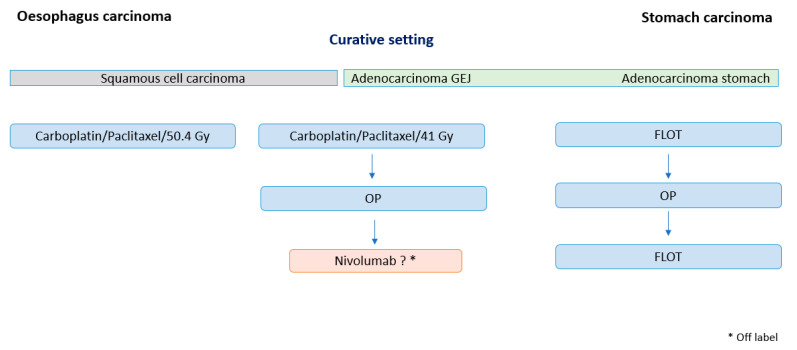
Overview of curative therapy regimes of oesophageal and gastric cancer.

Recent results of the randomised, multi-national, double-blind phase III study CheckMate-577 with adjuvant nivolumab or placebo in patients with lower oesophageal or gastroesophageal junction (GEJ) cancer following neoadjuvant chemoradiation therapy and R0-resection demonstrated superior efficacy of nivolumab regarding disease-free survival (DFS). The study included patients with residual disease in terms of non-pathological complete remission (≥ypT1, ≥ypN1). A total of 794 patients were randomised (532: nivolumab, 262: placebo); 70% of patients were suffering from adenocarcinoma and 30% of patients from squamous cell carcinoma. Presented in the presidential session of the ESMO (European Society for Medical Oncology) 2020, a significant benefit in DFS in patients treated with nivolumab was observed in the pre-specified interim analysis: adjuvant nivolumab showed a significant improvement of median DFS versus placebo (22.4 vs. 11.0 months, HR (hazard ratio) 0.69 (96.4% CI 0.56–0.86), *p* = 0.0003). Moreover, in all subgroups, there was a significant benefit in DFS in patients treated with nivolumab. The effect was more pronounced in ESCC compared with EAC (ESCC vs. placebo 29.7 months, unstratified HR 0.61 95% CI/EAC vs. placebo 19.4 months, unstratified HR 0.75 95% CI) [14]. Despite these encouraging results, it is of note that the follow up is only 24 months and no data for overall survival were presented. Implementing nivolumab in the adjuvant therapy of oesophageal cancer may, in our view, develop into a new standard in cancer treatment if further follow up confirms the benefit (Figure 2).

### 3.2. Palliative Setting: First-Line Therapy

Recently presented in the presidential session at ESMO 2020, the combination of pembrolizumab plus chemotherapy showed a significant improvement in the overall survival in patients with ESCC and EAC versus chemotherapy alone. In the KEYNOTE-590 trial, a randomised international double-blind phase III study of pembrolizumab plus chemotherapy (cisplatin + 5-FU) versus chemotherapy alone in 749 patients with locally advanced or metastatic oesophageal cancer (including Siewert type 1 adenocarcinoma of the esophago-gastric junction) were randomised 1:1 with 73% ESCC and 25% EAC patients. Independent from CPS (combined positivity score) and tumour histology, there was a significant benefit in OS (overall survival) in the combination group of pembrolizumab plus chemotherapy (OS all patients (pts) 12.4 vs. 9.8 months, HR 0.73 (95% CI 0.62–0.86, *p* < 0.0002); PFS (progression-free survival) all pts 6.3 vs. 5.8 months, HR 0.65 (95% CI 0.55–0.76)). In particular, ESCC patients with CPS ≥ 10 benefitted most from the combination of immune checkpoint inhibition and chemotherapy (median OS 13.9 vs. 8.8 months, HR 95% CI = 0.57 (0.43–0.75), *p* = 0.012). In this subgroup, 55% of patients treated with the combination therapy were alive after 12 months versus 34% treated with chemotherapy alone [15]. The CPS score seems to be decisive for response in subgroups. Adenocarcinomas seem to have a similar benefit from the addition of pembrolizumab compared to squamous cell carcinoma, but the confidence interval for adenocarcinoma is crossing 1 (HR 0.74 (0.54–1.02) versus 0.72 (0.60–0.88) in ESCC). It is of note that no separate Kaplan Meier graph was presented for adenocarcinomas. Keeping in mind the negative results for pembrolizumab plus chemotherapy in the KEYNOTE-062 trial presented below, which included adenocarcinoma of the stomach and esophago-gastric junction, in our view the overall effect for adenocarcinoma is less convincing compared to squamous cell oesophageal carcinoma.

In our view, at least for squamous cell carcinoma these data support the implementation of pembrolizumab in combination with chemotherapy as a new treatment in the palliative first-line setting of advanced oesophageal cancer.

### 3.3. Palliative Setting: Second-Line Therapy

The randomised phase III KEYNOTE-181 trial investigated the effect of pembrolizumab monotherapy versus chemotherapy of the investigator’s choice (paclitaxel, docetaxel, irinotecan) in patients with advanced or metastatic ESCC (64%) or EAC (36%) with progression after one prior chemotherapy independent of PD-L1 expression [16]. The primary endpoint was split and comprised OS in all patients, ESCC patients and PD-L1 CPS ≥ 10 patients. The median OS with pembrolizumab was 7.1 versus 7.1 months in all patients (HR 0.89, 95% CI 0.75–1.05, *p* = 0.0560, non-significant) and 8.2 versus 7.1 months in ESCC patients ((HR 0.78, 95% CI 0.63–0.96, *p* = 0.0095, non-significant), the significance margin due to a split primary endpoint was *p* < 0.0077), respectively. There was a significant improvement of OS in EAC and ESCC patients with PD-L1 CPS ≥ 10 (median OS 9.3 vs. 6.7 months, HR 0.69, 95% CI 0.52–0.93, *p* = 0.0074, significant). The OS benefit was most pronounced in the subgroup of ESCC with CPS ≥ 10. Based on these results, pembrolizumab is already approved by the FDA in the USA for second-line treatment of patients with ESCC and PD-L1 CPS ≥ 10.

**Figure 3:**
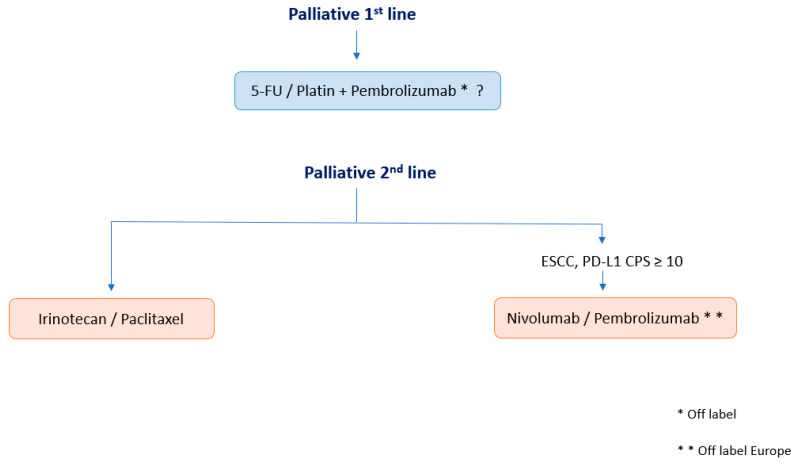
Overview of a possible algorithm for palliative therapy regimes in oesophageal cancer.

On the basis of the efficacy data of nivolumab versus chemotherapy in patients with ESCC in the ATTRACTION-03 trial, nivolumab has been approved in Asia, the USA, and recently in Europe in November 2020. This randomised, multicenter phase III trial included 419 patients suffering from advanced ESCC refractory or intolerant to previous chemotherapy to receive either nivolumab monotherapy or chemotherapy (paclitaxel or docetaxel). Independent from the PD-L1-expression rate, there was a significant benefit in the OS of nivolumab compared with chemotherapy (median OS 10.9 vs. 8.4 months, HR 95% CI: 0.77 (0.62–0.96), *p* = 0.019). After 12 months, 47% of patients were still alive in the nivolumab arm versus 34% in the chemotherapy arm [17]. As a result, nivolumab serves as a new second-line treatment option for these patients. In our view, due to the results of KEYNOTE-181 and ATTRACTION-03, checkpoint inhibition has a definite role in the palliative treatment of squamous cell carcinoma of the oesophagus. We consider the level of PD-L1 expression (CPS) a valid biomarker to help estimate the chance of benefit. An overview of possible implementation of checkpoint inhibitors into the palliative treatment of oesophageal cancer from the authors’ view is presented in Figure 3.

### 3.4. Summary Oesophageal Cancer

1. In the curative setting, nivolumab prolonged DFS in patients with ESCC and EAC across all subgroups, with a more pronounced effect in ESCC. OS data are awaited. Nivolumab may become a new standard of care in adjuvant therapy of oesophageal cancer (CHECKMATE-577).
2. Based on KEYNOTE-590 results, implementation of pembrolizumab in combination with chemotherapy, especially in squamous cell carcinoma, might develop into a new treatment in palliative first-line therapy of advanced oesophageal cancer, especially ESCC.
3. Improvement of OS by pembrolizumab in patients with advanced ESCC, especially with PD-L1 CPS ≥ 10, led to FDA’s approval of pembrolizumab in the second-line setting in the USA (KEYNOTE-181).

Moreover, independent of the PD-L1 expression status, nivolumab significantly increases OS in patients with advanced oesophageal cancer (ATTRACTION-03). Nivolumab monotherapy serves as new second-line treatment option in patients with advanced or metastatic ESCC after progression of first-line therapy with approval for nivolumab in Asia, the USA, and Europe.

## 4. Immune Checkpoint Inhibition in the Treatment of GEJ and Gastric Cancer (GC)

### 4.1. Curative Setting

In the perioperative therapy of gastric cancer, checkpoint inhibitors are only applied within clinical trials. Atezolizumab, a selective anti-PD-L1 antibody, was investigated in the randomised multicenter phase II trial “DANTE” from the AIO (Arbeitsgemeinschaft Internistische Onkologie) in patients with resectable, localised adenocarcinoma of the GEJ or stomach which just finished accrual. Patients were randomised equally in the experimental arm with combination therapy of atezolizumab and chemotherapy (FLOT regimen: 5-FU, folinic acid, oxaliplatin, docetaxel) and FLOT only [18]. The primary endpoint was progression/disease free survival defined as the time from randomisation to disease progression or relapse. The recently updated safety data of the study assessed atezolizumab plus FLOT as feasible and safe in the perioperative setting; efficacy results are awaited [19].

### 4.2. Palliative Setting: First-Line Therapy

In the KEYNOTE-062 trial, pembrolizumab monotherapy showed non-inferiority compared to chemotherapy (cisplatin + 5-FU/capecitabine) in patients with PD-L1 positive (combined positivity score (CPS) ≥ 1) advanced adenocarcinoma of the gastrooesophageal junction or stomach (HR 0.91, 95% CI 0.69–1.18). A total of 763 patients (30% GEJ, 69% stomach cancer) were randomised in three arms with either pembrolizumab monotherapy, chemotherapy plus placebo, or a combination of pembrolizumab plus chemotherapy (cisplatin/5-FU or capecitabine). Whereas pembrolizumab monotherapy was non inferior to chemotherapy in patients with CPS ≥ 1 (10.6 vs. 11.1 months, HR 0.74 (95% CI 0.74–1.10), *p* = 0.162) (primary endpoint), pembrolizumab monotherapy prolonged OS in patients with PD-L1 CPS ≥ 10 (median OS 17.4 vs. 10.8 months, HR 0.69, 95% CI 0.49–0.97). However, illustrated in the crossing survival curves of OS, this subgroup also comprised patients who died faster under pembrolizumab compared to chemotherapy, whereas another group of patients lived longer under treatment with pembrolizumab.

With regard to the combination of pembrolizumab and chemotherapy, patients either with PD-L1 CPS ≥ 1 (OS 12.5 vs. 11.1 months, HR 0.85 (95% CI 0.7–1.03), *p* = 0.046) or PD-L1 CPS ≥ 10 (12.3 vs. 10.8 months, HR 0.85 (0.62–1.17), *p* = 0.158) did not show superior OS when adding pembrolizumab to chemotherapy compared to chemotherapy alone [20]. Different explanations for this are discussed: the choice of the chemotherapy backbone (less immunogenicity of cisplatin compared to oxaliplatin), a T-cell suppressive effect of continuous infusion of 5-FU, or a yet unknown selection bias or statistical chance were all a matter of debate. Remarkably, patients with microsatellite instability (MSI)-high tumours specifically benefitted from pembrolizumab versus chemotherapy with prolongation of OS as discussed in detail below.

In our view, KEYNOTE-062 shows a clear benefit of pembrolizumab in a subgroup of patients. However, even CPS ≥ 10 as a biomarker could not separate completely between patients benefitting from pembrolizumab monotherapy and those who do better when treated with upfront chemotherapy.

Presented at ASCO GI 2020, the randomised global phase III trial “JAVELIN-100 Gastric study” investigated the effect of avelumab (PD-L1 checkpoint inhibitor) maintanance therapy after at least stable disease for 12 weeks by first-line therapy with 5-FU and platin versus continuation of first-line chemotherapy in 499 patients with advanced, metastasised adenocarcinoma of the GEJ and stomach. Despite good tolerabilty, there was no advantage in the overall survival (primary endpoint) and duration of response by using avelumab as a maintenance therapy. An exploratory analysis concerning the PD-L1 CPS score indeed showed a trend for prolonged overall survival [21]. Nivolumab could recently show a significant OS improvement in a landmark trial. The randomised CheckMate-649 trial, the largest international phase III trial with 1581 patients suffering from locally advanced or metastastic Her2/neu negative adenocarcinoma of the gastroesophageal junction or stomach evaluated the effect of nivolumab plus chemotherapy (XELOX (capecitabine plus oxaliplatin) or FOLFOX (5-FU, folinic acid, oxaliplatine) versus chemotherapy alone as the first-line regime. The results of the prespecified interim analysis of OS and PFS in this study were presented at ESMO 2020 also in the presidential session. The combination of nivolumab plus 5-FU/oxaliplatin improved OS and PFS in patients with PD-L1 CPS ≥ 5 (primary endpoint, *n* = 955 patients, 60%) significantly. Improvement of median OS was 14.4 months versus 11.1 months (HR 98% CI = 0.71 (0.59–0.86), *p* < 0.0001). Differences were also statistically significant for all patients with PD-L1 CPS ≥ 1 (HR = 0.77, *p* = 0.0001) and for all randomly assigned patients irrespective of their PD-L1 CPS score (HR = 0.80, *p* = 0.0002). Because 70% of all patients have CPS ≥ 1 and 60% CPS ≥ 5, the positive results for these groups are likely to be driven by the CPS ≥ 5 population. In the group of patients with PD-L1 CPS ≥ 5, the median progression-free survival was 7.7 versus 6.0 months, respectively (HR 98% CI = 0.68 (0.56–0.81), *p* < 0.0001) [22]. In particular, patients with CPS ≥ 5 and MSI (microsatellite instability)-high tumours seem to benefit from the combination of nivolumab and chemotherapy as presented in detail in the biomarker paragraph below. Based on the results of this trial, the implementation of nivolumab as a first-line therapy option for advanced GEJ or stomach cancer with PD-L1 CPS ≥ 5 is expected.

The Asian phase II–III trial ATTRACTION-04 supported these data, as it investigated nivolumab in combination with chemotherapy (CAPOX or S-1 plus Oxaliplatin) versus nivolumab alone in patients with advanced human epidermal growth factor receptor-2 (HER2)-negative GEJ or gastric cancer irrespective of PD-L1 expression. This showed a significant improvement of PFS in the combination therapy, whereas OS was not significantly altered [23]. In contrast to CHECKMATE-649, the primary endpoints were designed for all-comers without regard to CPS scores. In ATTRACTION-04, 66% of patients received further lines of treatment compared to 38% in CHECKMATE-649, which may explain the different OS effect observed in these two trials.

In our view, data to support the implementation of nivolumab together with chemotherapy as a new first-line treatment option for adenocarcinoma of the stomach and GEJ with a CPS ≥ 5 are very strong and will probably change our future treatment algorithm.

### 4.3. Palliative Setting: Second-Line Therapy

Within the KEYNOTE-061 trial, 592 patients with progressive GEJ or stomach cancer were randomised in pembrolizumab monotherapy versus paclitaxel, irrespective of PD-L1 expression. This trial did not reach its primary endpoint (superiority in OS in patients with PD-L1 CPS ≥1, *n* = 397). However, the data reflect the dependence of the efficacy of pembrolizumab on the grade of positive PD-L1 CPS. Thus, for patients with PD-L1 CPS < 1, pembrolizumab treatment was less efficient compared with paclitaxel. Survival curves overlap at PD-L1 CPS ≥ 1, but demonstrate superiority of pembrolizumab in the subgroup of PD-L1 CPS ≥ 10. Overall, patients with ECOG (Eastern Cooperative Oncology Group) performance status 0 seem to benefit more from pembrolizumab instead of paclitaxel [24]. At ASCO 2020, the role of tumor mutational burden (TMB) for efficacy of checkpoint inhibition was discussed. TMB might act as a predictive marker, whereby there is no correlation of TMB and PD-L1 CPS [25,26].

Moreover, the effect of avelumab, the selective PD-L1 inhibitor, in patients with advanced GEJ or gastric cancer is analysed in the single-arm phase II “RAP” trial. This study investigates the incorporation of PD-L1 blockade by avelumab in the second-line setting in combination with the paclitaxel and ramucirumab [27]. Combining immunotherapy with VEGF-directed treatment and chemotherapy is supposed to increase the immunogeneity of tumour cells [28]. Recruitment of patients is completed, and the first data of efficacy are expected.

### 4.4. Palliative Setting: Third-Line Therapy

The administration of pembrolizumab independent from PD-L1 expression status was investigated in the third-line setting in the multicohort nonrandomised phase II trial KEYNOTE-059. Pembrolizumab showed efficacy especially in PD-L1 positive tumours (PD-L1 expression ≥ 1%) with a better overall response compared to PD-L1 negative tumours (ORR (overall response rate) 15.5% vs. 6.4%, respectively; median OS 5.8 vs. 4.6 months, primary endpoint). In PD-L1-positive responders, the duration of response (DOR) was prolonged, respectively (DOR all patients 8.4 months, PD-L1-positive patients 16.3 months) [29]. Including these data, pembrolizumab is approved in the USA for the second-line treatment of advanced PD-L1-positive chemorefractory gastric cancer.

Nivolumab was investigated in the Asian ATTRACTION-02 trial in progressive gastric cancer after two therapy lines including 493 patients with advanced GEJ or stomach cancer independent from PD-L1 expression status. Patients treated with nivolumab versus placebo showed an overall response of 11.4% with improvement of overall survival (median OS 5.3 months vs. 4.1 months; HR 0.63, 95% CI 0.51–0.78; *p* < 0.001, primary endpoint). After one year, 26.2% of patients treated with nivolumab were still alive compared with 10.9% of patients treated with placebo [30]. These results lead to approval of nivolumab in the third-line therapy of GEJ and gastric cancer in Asia.

**Figure 4:**
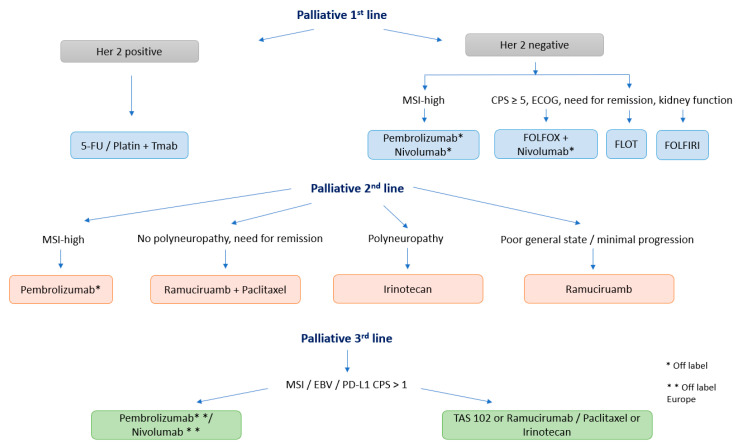
Overview of a possible algorithm for palliative therapy regimes in gastric cancer.

Due to innovative therapeutic options including immune checkpoint inhibition, gastric cancer patients with progression after two palliative therapy lines in good performance status are able to be treated with well tolerable antibodies with the aim of improving quality of life and survival. A possible algorithm for palliative therapy regimens in gastric cancer from the author’s view is presented in Figure 4.

### 4.5. Role of Combination of Immune Checkpoint Inhibitors and Anti-HER2 Therapy in GEJ/Stomach Cancers

In patients with HER2 (human epidermal growth factor receptor-2)-positive metastatic gastric cancer, the addition of trastuzumab to first-line chemotherapy improves overall survival. In the TOGA trial, in HER2-positive patients with advanced GEJ/GC (*n* = 446, 12% of total population), the combination of trastuzumab plus chemotherapy (Cisplatin + 5-FU/Capecitabine) improved median overall survival by 4.2 months (HR = 0.65, 95% CI: 0.52–0.83) [31]. HER2-positivity is defined as immunohistochemical expression of level 3+ or level 2+ combined with positive FISH verification of HER2 gene amplification. Adding trastuzumab to chemotherapy in HER2-positive patients with advanced GEJ/stomach cancer in first-line therapy became an international standard of care and is recommended in ESMO-guidelines [3].

Until now, few clinical trials are investigating the combinatory effect of targeted HER2 therapy with immune checkpoint inhibition and cytotoxic chemotherapy in GEJ cancer and gastric cancer. Possible contributers to synergistic mechanisms of anti-HER2 and anti-PD-1-based therapies have been under investigation. In early HER2-positive breast cancer, tumour core biopsies from two multicenter trials at baseline and after exposure to trastuzumab or nab-paclitaxel were investigated for correlation of gene expression profiles and immune signatures with response to therapy. Treatment with trastuzumab significantly increased gene expression of PD-1 and immune cell activity, as well as PD-1-positivity by immunohistochemistry in HER2-enriched tumours, which could finally increase the efficacy of pembrolizumab [32,33]. Additionally, PD-L1 is upregulated after the administration of trastuzumab in a breast cancer mouse model [34]. Further, trastuzumab may prime antitumor immune responses by the induction of antibody-dependent cell-mediated cytotoxicicty resulting in an enhanced production of tumour antigens and a possible response to immune therapy [35].

The interplay of the antitumor immune reaction by anti-HER2 therapy and cytotoxic chemotherapy with immune checkpoint blockade has been analysed in a clinical setting in patients with advanced GEJ/gastric cancer with promising activity. The combination of pembrolizumab, trastuzumab, and chemotherapy (oxaliplatin/cisplatin plus cabecitabine/5-FU) was investigated in a single-arm phase II trial in 37 patients with HER2-positive metastatic oesophagogastric cancer. The study included 38% of patients with advanced oesophageal cancer, 32% with GEJ, and 30% gastric cancer patients. After 6 months, 26/37 patients were progression free (70% (95% CI 54–38), primary endpoint). The overall survival rate (OSR) after 12 months was 80% (95% CI 68–95) and treatment duration was 10 months (IQR 5.7–13.7). Initially, all patients showed tumor shrinkage, including two patients with evaluable but non-measurable disease by RECIST 1.1 criteria (stable disease as best response) [33]. This study represents a safe combination of pembrolizumab plus trastuzumab and chemotherapy with promising effects in the first-line chemotherapy of gastro-oesophageal cancer patients.

With respect to nivolumab, the efficacy of the combination of the CTLA-4 (anti-cytotoxic T-lymphocyte-associated antigen 4) inhibitor ipilimumab or FOLFOX chemotherapy (5-FU, folinic acid, oxaliplatin) with nivolumab and trastuzumab in the first-line setting is being investigated in 97 previously untreated HER2-positive patients with advanced or metastatic GEJ adenocarcinoma in the phase II INTEGA trial. Patients are randomised to receive either trastuzumab, nivolumab and ipilimumab (arm A), or trastuzumab, nivolumab and mFOLFOX (arm B). Clinical benefit will be measured by OS (primary endpoint), PFS, and ORR (secondary endpoints). The recruitment is completed and the first results are expected in October 2021 [36].

Other trials also investigating combinations of HER 2 blockade and checkpoint inhibition are still recruting.

### 4.6. Summary GEJ/Gastric Cancer

1. In the first-line setting, patients with PD-L1 CPS ≥ 5 benefit from the combination of nivolumab and chemotherapy (CHECKMATE-649). Pembrolizumab also shows efficacy in combination with chemotherapy in patients with Siewert Type 1 GEJ adenocarcinoma (KEYNOTE-590) and is beneficial as a monotherapy in a subgroup of gastric cancer patients with PD-L1 CPS ≥ 10 and MSI-high tumors (KEYNOTE-062).
2. Response to immunotherapy seems to be dependent on the grade of PD-L1 CPS positivity with the strongest effect in OS in PD-L1 CPS ≥ 10 patients.
3. In the third-line setting, nivolumab prolonged OS in patients with advanced GEJ/gastric cancer after progression of at least two prior therapy regimes and is approved for third-line treatment in Asia (ATTRACTION-02 trial). Similar results were achieved with pembrolizumab in Caucasian patients in the single-arm phase II KEYNOTE-059 trial, resulting in FDA approval of pembrolizumab in the USA in patients with chemotherapy refractory PD-L1 positive GEJ and gastric cancer. So far, there is no approval for checkpoint inhbition in gastric cancer in Europe.
4. Augmenting the effect of anti-HER2-targeted therapy in combination with immune checkpoint blockade is under investigation.
5. In particular, MSI-high patients seem to benefit from anti-PD-L1/PD-1-targeted therapy.

**Table 1:** Overview of immune checkpoint inhibitor clinical trials in oesophago-gastric cancer.

Entity	Author	Ref.	Trial	Phase	Treatment	N	Localisation	Histology	PD-L1 Score	Results (Primary Enpoint, Median)	Results (Further Analysis, Median)
Oesophageal cancer											
Curative	Kelly et al.	[14]	Checkmate-577	III	Adjuvant Nivo vs. placebo	794	Oe/GEJ	ESCC (30%), EAC (70%)	all comers	DFS 22.4 vs. 11 months (HR 0.69 (95% CI 96.4% CI 0.56–0.86), *p* = 0.0003	
First-line	Kato et al.	[15]	Keynote-590	III	Pembro + Cis/5-FU vs. Cis/5-FU alone	749	Oe/GEJ	ESCC (73%), EAC (27%)	all comers	OS all 12.4 vs 9.8 months (HR, 0.73, 95% CI, 0.62–0.86), *p* < 0.0001	ORR all 45% vs. 29.3%, *p* < 0.0001
										OS ESCC 12.6 vs. 9.8 months (HR 0.72; 95% CI, 0.60–0.88), *p* = 0.0006	
										OS PD-L1 CPS ≥ 10: 13.5 vs. 9.4 months (HR 0.62; 95% CI, 0.49–0.78), *p* < 0.0001	
										OS ESCC PD-L1 CPS ≥10 median 13.9 vs. 8.8 mo; (HR 0.57; 95% CI, 0.43–0.75)	
										PFS all pts 6.3 vs. 5.8 months (HR 0.65; 95% CI, 0.55–0.76), *p* < 0.0001	
										PFS ESCC 6.3 vs. 5.8 months (HR 0.65; 95% CI, 0.54–0.78), *p* < 0.0001	
										PFS CPS ≥10: 7.5 vs. 5.5 months (HR 0.51; 95% CI, 0.41–0.65) *p* < 0.0001	
Second-line	Kojima et al.	[16]	Keynote-181	III	Pembro vs. Pacli/Doce/Irino	628	Oe/GEJ	ESCC (64%), EAC (36%)	all comers	OS all 7.1 vs. 7.1 months (HR 0.89; 95% CI, 0.75–1.05), *p* = 0.0560	
										OS ESCC 8.2 vs. 7.1 months (HR 0.78; 5% CI, 0.63–0.96), *p* = 0.0095	
										OS CPS ≥ 10: 9.3 vs. 6.7 months (HR 0.69; 95% CI, 0.52–0.93), *p* = 0.0074	
	Kato et al.	[17]	Attraction-03	III	Nivo vs. Pacli/Doce	419	Oe	ESCC	all comers	OS 10.9 vs. 8.4 months (HR 0.77, 95% CI 0.62–0.96), *p* = 0.019	
GEJ/Stomach cancer											
Curative	Al-Batran et al.	[18]	Dante	II	Atezo + FLOT vs. FLOT alone	295	GEJ/GC	Adenocarcinoma	all comers	DFS (awaited)	Rate of pathological regression (awaited)
First-line	Moehler et al.	[22]	Checkmate-649	III	Nivo + XELOX/FOLFOX vs. Nivo + Ipi vs. Chemo alone	1581	GEJ/GC	Adenocarcinoma	all comers	OS PD-L1 CPS ≥ 5: 14.4 vs. 11.1 months, HR 0.71 (98.4% CI 0.59–0.86), *p* < 0.0001	OS all 13.8 vs. 11.6 months (HR 0.80; 99.3% CI 0.68–0.94), *p* = 0.0002
									(60% PD-L1 CPS ≥ 5)	PFS PD-L1 CPS ≥ 5: 7.7 vs. 6.0 months; HR (98% CI 0.56–0.81), *p* < 0.0001	OS PD-L1 CPS ≥ 1: 14.0 vs. 11.3 months, HR 0.77 (99.3% CI 0.64–0.92), *p* = 0.0001
											PFS all 7.7 vs. 6.9 months; HR 0.77 (95% CI 0.68–0.87), *p* > 0.05
											PFS PD-L1 CPS ≥1: 7.5 vs. 6.9 months; HR 0.74 (95% CI 0.65–0.85), *p* > 0.05
	Shitara et al.	[20]	Keynote-062	III	Pembro vs. Cis/5-FU or Cape; (p/p + c/placebo + c)	763	GEJ/GC	Adenocarcinoma	PD-L1 CPS ≥ 1	OS PD-L1 CPS ≥1: 10.6 vs. 11.1 months; HR 0.74 (95% CI 0.74–1.10), *p* = 0.162, *p* non inferior to c	
										OS PD-L1 CPS ≥10: 17.4 (p) vs 10.8 months (c); HR 0.69 (95% CI 0.49–0.97)	
										OS PD-L1 CPS ≥1: 12.5 (p + c) vs. 11.1 months (c); HR 0.85 (95% CI 0.7–1.03), *p* = 0.046, p + c not superior	
										OS PD-L1 CPS ≥10: 12.3 (p + c) vs. 10.8 months (c): HR 0.85 (0.62–1.17), *p* = 0.158, p + c not superior	
										PFS PD-L1 CPS ≥1: 6.9 (p + c) vs. 6.4 months (c): HR 0.84 (0.70–1.02), *p* = 0.04	
Second-line	Shitara et al.	[24]	Keynote-061	III	Pembro vs. Pacli	592	GEJ/GC	Adenocarcinoma	all comers (67% PD-L1 CPS ≥ 1)	OS PD-L1 CPS ≥1: 9.1 vs. 8.3 months; HR 0.81 (95% CI 0.66–1.00), *p* > 0.05	OS PD-L1 CPS ≥ 5: 10.4 vs. 8.3 months; HR 0.72 (95% CI 0.53–0.99), *p* > 0.05
										PFS PD-L1 CPS ≥1: 1.5 vs. 4.1 months; HR 1.27 (95% CI 1.03–1.57)	OS PD-L1 CPS ≥ 10: 10.4 vs. 8.0 months; HR 0.69 (95% CI 0.46–1.05), *p* > 0.05
	Thuss-Patience et al.	[27]	RAP	II	Ram + Avel + Pacli, single arm	60	GEJ/GC	Adenocarcinoma	all comers	OSR at 6 months (awaited)	OS, OSR at 12 months, PFS, DoR (awaited)
	Moehler et al.	[21]	Javelin Gastric-100	II	Avel vs. maintainance 1st line	499	GEJ/GC	Adenocarcinoma	all comers	OS all 10.4 vs. 10.9 months; HR 0.91 (95% CI 0.74–1.11), *p* = 0.1779;	ORR 13.3% (95% CI 9.3–18.1) vs. 14.4% (95% CI 10.3–19.4)
										OS PD-L1 + (n = 54): HR 1.13 (95% CI 0.57–2.23)	DOR (12 months): 62.3% (95% CI 40.9–77.9) vs. 28.4% (95% CI 13.2–45.7)
Third-line	Kang et al.	[30]	Attraction-02	III	Nivo vs. placebo	493	GEJ/GC	Adenocarcinoma	all comers	OS 5.3 vs. 4.14 months; HR 0.63 (95% CI 0.51–0.78), *p* < 0.0001)	
	Fuchs et al.	[29]	Keynote-059	II	Pembro mono	259	GEJ/GC	Adenocarcinoma	all comers	ORR PD-L1+/-: 15.5% (95% CI, 10.1–22.4%) vs. 6.4% (95% CI 2.6%–12.8%)	Response duration PD-L1+/-: 16.3 months (95% CI 1.6–17.3) vs. 6.9 months (95% CI 2.4–7.0)
HER2 positive cancer											
First-line	Tintelnot et al.	[36]	Intega	II	Ipi/FOLFOX + Nivo + Tmab	97	GEJ	Adenocarcinoma	all comers	OS (awaited)	PFS, ORR (awaited)
	Janjigian et al.	[33]	Pembro + Tmab	II	Pembro + Tmab + chemo (Oxali/Cis + Cape/5-FU) Single-arm	37	Oe/GEJ/GC	Adenocarcinoma	all comers	PFS 6 months: 70% (95% CI 54–83); mPFS 13.0 months (95% CI 8.6-NR)	OS 27.3 months (95% CI 18.8-NR), 12 months OSR: 80% (95% CI 68–95)
											Treatment duration: 10 months (IQR 5.7–13.7)

Nivo = Nivolumab, Ipi = Ipilimumab, Pembro = Pembrolizumab, Atezo = Atezolizumab, Avel = Avelumab, Ram = Ramucirumab, Tmab = Trastuzumab, Pacli = Paclitaxel, Doce = Docetaxel, Irino = Irinotecan, Cis = Cisplatin, Cape = Capecitabine, Oxali = Oxaliplatin, Oe = Oesophagus, GEJ = gastrointestinal junction, GC = gastric cancer, OS = overall survial, ORR = overall response rate, PFS = progression free survival, PFSR = progression free survival rate, DFS = disease-free survival, HR = Hazard ratio, IQR = interquartile range, NR = not reached.

## 5. Role of Biomarkers in Oesophago-Gastric Cancers (PD-L1 CPS, EBV, MSI-High)

### 5.1. PD-L1 CPS (PD-L1 Combined Positivity Score) in GEJ/Gastric Cancers

Since the comprehensive molecular characterisation of gastric cancer into the four genomic subtypes of chromosomally instable tumours, Epstein–Barr virus-infected tumors, microsatellite instable tumors, and genomically stable tumours in 2014, rapid progression in developing individual therapeutic approaches to specified molecular targets has been made [37].

PD-L1 CPS shows encouraging potential as predictive marker for immune response to anti-PD-1/PD-L1 therapy in squamous cell carcinoma of the oesophagus, as well as in adenocarcinomas of the GEJ and stomach.

There are organ-specific differences in PD-1 histological diagnosis including different scoring algorithms which are specific to tumour entities and clinical decisions. Whereas in NSCLC, melanoma, and head-neck-tumors, the TPS (“tumour proportion score”) with consideration of exclusively membranous PD-L1 staining in tumour cells is established, the PD-L1 CPS-scoring becomes the standard analysis in urothelial and GEJ/stomach cancer samples. PD-L1 CPS thereby includes the total of positive stained tumour cells (linear membranous staining) and positive stained mononuclear immune cells (including macrophages, lymphocytes, dendritic cells with simultaneous consideration of membranous and cytoplasmatic staining) divided by the basic population of tumour cells [38]. Current cut-offs for the PD-L1 CPS vary between different tumour entities. In GEJ and gastric cancers, the PD-L1 CPS cut-off for best prediction of efficacy still has to be defined. PD-L1 CPS ≥ 1 defines PD-L1 positivity. In pembrolizumab trials, CPS ≥ 10 showed best discrimination; in nivolumab trials, a cut-off of CPS 5 was used.

As presented in detail above, various studies of oesophageal and gastric cancer reveal benefits in overall survival from immunotherapy with pembrolizumab in ESCC patients (e.g., KEYNOTE-059/181) and GEJ/stomach cancer patients (e.g., KEYNOTE-059/062/061), as well as nivolumab (e.g., CHECKMATE-649). Remarkably, response to immunotherapy in terms of prolonged overall survival seems to be dependent on the amount of PD-L1 CPS positivity: the higher the CPS score, the higher the benefit in OS: patients with CPS ≥ 5 versus CPS ≥ 1 (nivolumab: CHECKMATE-649), CPS ≥ 10 with OS benefit vs. CPS ≥ 1 (pembrolizumab: KEYNOTE-062/061). This is confirmed in a recent survey analysing patients with CPS ≥ 10 treated with pembrolizumab [39].

### 5.2. EBV-Positive Gastric Cancers

Over 90% of the world population is latent infected throughout life with the Epstein–Barr Virus (EBV), accounting for 9% in all gastric adenocarcinomas [40]. Analysis of the TCGA (The Cancer Genome Atlas) cohort shows evidence of EBV in overall gastric cancer patients of 8.2% with predominance in male patients (81%), young patients, gastric fundus, or body independent from histological subtype [41,42]. EBV-positive gastric cancers reveal a higher expression of immune checkpoint genes (e.g., PD-1, CTLA-4) and higher histological lymphocytic infiltration compared with MSS(microsatellite-stable) tumours [43]. The role of EBV-positive gastric cancers as a possible predictive marker for response to targeted immunotherapy is being investigated in clinical trials. There were impressive responses to pembrolizumab in the subgroups of patients with EBV-positive tumors, mutually exclusive. Observed in the molecular characterisation of 61 patients from a prospective phase II trial with pembrolizumab as salvage therapy in metastatic gastric carcinoma, the overall response rate was 100% in EBV-positive tumors (*n* = 6). In the same analysis 7 patients with MSI high tumours were identified, mutually exclusive to the EBV-positive tumours. A total of 85.7% of the MSI-high tumors responded with tumour shrinkage. From 55 patients with an available positive PD-L1 CPS score (PD-L1 CPS ≥ 1%), the ORR was significantly higher in PD-L1 positive gastric cancer compared with PD-L1 negative cancers (50% vs. 0%, *p* < 0.001) [44].

### 5.3. MSI-High GEJ/Gastric Cancers

High mutational burden has been considered to accomplish a continuous clinical benefit with anti-PD-1/PD-L1 treatment in different types of tumours, including gastrointestinal cancers [13,45,46].

In particular, tumors with microsatellite instability (MSI), distinctive of deficient mismatch repair, exhibit a high mutational load by creating tumor neoantigens and thereby are targeted, in particular, by immune response mechanisms [37].

The subgroup of MSI-high tumors was analysed for efficacy in the pembrolizumab trial for first-line treatment (35 patients, KEYNOTE-062 trial), as well as in second-line treatment (27 patients, KEYNOTE-061 trial). In KEYNOTE-062, patients with PD-L1 CPS ≥ 1 with MSI-high tumors specifically benefit from pembrolizumab versus chemotherapy with prolongation of overall survival after 12 months from 47% to 79% (HR 0.29, 95% CI) [20].

In second-line treatment with pembrolizumab versus paclitaxel in the KEYNOTE-061 trial, MSI-high cancer patients showed a benefit in 12-months overall survival of 25% with paclitaxel (95% CI 0.13–1.31) and 70% with pembrolizumab (95% CI 0.42) [24,25].

Furthermore, the combination of nivolumab and chemotherapy in the first-line treatment of PD-L1 CPS ≥ 5 GEJ cancer/gastric cancer patients also showed a pronounced benefit in OS in the MSI-high subgroup (HR 0.33, 09% CI; CHECKMATE-649 trial) [22].

### 5.4. Summary Biomarkers in Oesophago-Gastric Cancers

1. The specific molecular subsets of patients with PD-L1 CPS, EBV or MSI-high status, qualify as potential predictive biomarkers and may identify individual patients who benefit most from immunotherapy in terms of prolonged overall survival.
2. In the authors’ view, especially patients with advanced MSI-high and PD-L1 CPS-positive cancers should obtain access to immune checkpoint inhibition in various palliative therapy settings.

## 6. Conclusions

At present we witness an encouraging development in gastro-oesophageal cancers by targeting immune checkpoints. Impressive phase II and landmark phase III trials investigated the effect of the anti-PD-1 antibodies nivolumab and pembrolizumab in different therapy sequences with benefits in overall survival. The PD-L1 CPS score and the microsatellite status prove to be good predictive markers for predicting response to checkpoint inhibition. In oesophageal cancer, the application of nivolumab as an adjuvant treatment after radiochemotherapy and resection showed a prolongation of DFS and has the potential to develop as a new standard when more data become available. In the first line setting, pembrolizumab added to standard chemotherapy could improve OS and may change the treatment algorithm, especially for oesophageal squamous cell carcinoma. In second-line treatment for squamous cell disease, nivolumab and pembrolizumab (for CPS ≥ 10) proved to be superior to chemotherapy. In gastric cancer, pembrolizumab and nivolumab show high activity in MSI-high tumors. Pembrolizumab shows CPS dependent efficacy in first- and second-line trials and non-inferiority to chemotherapy (first-line), benefitting a subgroup of patients. Nivolumab improved significantly OS in the first-line when added to chemotherapy and may develop as a new standard for the first-line treatment of gastric and GEJ adenocarcinoma with a PD-L1 CPS ≥ 5. In chemotherapy refractory disease, nivolumab also shows benefits compared to best supportive care.

In conclusion, randomised phase III trials demonstrate a consistent benefit of checkpoint inhibition in a subgroup of gastro-oesophageal cancers. MSI-high, PD-L1 CPS, and EBV may serve as predictive biomarkers, but further refinement and harmonisation of biomarker scoring is warranted. In view of the high costs of checkpoint inhibitors, cost effectiveness analyses are awaited before widespread adaptation of these agents. Nevertheless, checkpoint inhibitors will develop as a new treatment tool with a definite role in squamous cell and adenocarcinoma of the oesophagus and stomach in the near future. Multiple trials are ongoing which further investigate the role of checkpoint inhibitors in combination regimens.

## Data Availability

Not applicable.
